# Gut microbiota of patients with different subtypes of gastric cancer and gastrointestinal stromal tumors

**DOI:** 10.1186/s13099-021-00403-x

**Published:** 2021-02-17

**Authors:** Virinder Sarhadi, Binu Mathew, Arto Kokkola, Tiina Karla, Milja Tikkanen, Hilpi Rautelin, Leo Lahti, Pauli Puolakkainen, Sakari Knuutila

**Affiliations:** 1 Faculty of Medicine, Department of Pathology, University of Helsinki, 00014 Helsinki, Finland; 2 Department of Computing, University of Turku, Turku, Finland; 3 The HUCH Gastrointestinal Clinic, University Central Hospital of Helsinki, Helsinki, Finland; 4 Thermo Fisher Scientific, Vantaa, Finland; 5 Department of Medical Sciences, Clinical Microbiology, Uppsala University, Uppsala, Sweden

**Keywords:** Diffuse gastric adenocarcinoma, Intestinal gastric adenocarcinoma, GIST, Gut microbiota

## Abstract

**Background:**

Gastric adenocarcinoma is associated with *H. pylori* infection and inflammation that can result in the dysbiosis of gastric microbiota. The association of intestinal microbiota with gastric adenocarcinoma subtypes or with gastric gastrointestinal stromal tumors (GIST) is however not well known. Therefore, we performed 16S rRNA gene sequencing on DNA isolated from stool samples of Finnish patients and controls to study differences in microbiota among different histological subtypes of gastric adenocarcinoma, gastric GIST and healthy controls.

**Results:**

We found that gut microbiota alpha diversity was lowest in diffuse adenocarcinoma patients, followed by intestinal type and GIST patients, although the differences were not significant compared to controls. Beta-diversity analysis however showed significant differences in microbiota composition for all subtypes compared to controls. Significantly higher abundance of *Enterobacteriaceae* was observed in both adenocarcinoma subtypes, whereas lower abundance of *Bifidobacteriaceae* was seen only in diffuse adenocarcinoma and of *Oscillibacter* in intestinal adenocarcinoma. Both GIST and adenocarcinoma patients had higher abundance of *Enterobacteriaceae* and lower abundance of *Lactobacillaceae* and *Oscillibacter* while lower abundance of *Lachnoclostridium, Bifidobacterium, Parabacteroides* and *Barnesiella* was seen only in the adenocarcinoma patients.

**Conclusions:**

Our analysis shows association of higher *Enterobacteriaceae* abundance with all types of gastric tumors. Therefore it could be potentially useful as a marker of gastric malignancies. Lower gut microbiota diversity might be indicative of poorly differentiated, invasive, advanced or aggressive tumors and could possibly be a prognostic marker for gastric tumors.

## Background

Gut microbiota has an important role in the maintenance of healthy gut, via its role in host metabolism, nutrient absorption, pathogen protection, immunity and local gut environment [reviewed in 1]. Composition of gut microbiota is mainly influenced by diet, ethnicity, and disease state [reviewed in 2]. *Helicobacter pylori (H. Pylori)* infection is strongly associated with gastric inflammation and with the carcinogenesis of gastric cancer. The most common type of gastric cancer (GC) is gastric adenocarcinoma, which is histologically grouped, according to Lauren’s classification, into two main subtypes; ‘diffuse’ and ‘intestinal’, with some cases exhibiting features of both diffuse and intestinal *i.e.* with ‘indeterminate’ or ‘mixed’ phenotype [3]. Both subtypes are associated with *H. pylori* infection, although it is more common in intestinal subtype [4]. However, the two subtypes have different carcinogenic pathway and pathogenesis. Intestinal adenocarcinoma is usually linked with a history of past inflammation of the stomach and is preceded by many premalignant stages, including intestinal metaplasia, while diffuse adenocarcinoma has poorly differentiated cell morphology and is often associated with poor prognosis and survival, compared to intestinal adenocarcinoma [5]. At molecular level, intestinal adenocarcinoma has more genetic imbalance, including microsatellite instability (MSI) and chromosomal instability [6, 7] than diffuse adenocarcinoma, which is genetically more stable but is associated with small-sized amplifications and mutations in E-cadherin gene (*CDH1*) [8, 9].

Gastrointestinal stromal tumors (GIST) are rare and are very different from gastric adenocarcinoma. They are commonly located in stomach, although they can also be present in other parts of the gastrointestinal tract. They originate from stromal cells and have *KIT* or *PDGRA* mutations [reviewed in 10].

Changes in gastric microbiota are seen in patients with gastric adenocarcinoma [reviewed in 11], however, very little is known about changes in intestinal microbiota of gastric adenocarcinoma and GIST patients. Since diffuse adenocarcinoma, intestinal adenocarcinoma and GIST of stomach differ in histology and pathology, we analyzed and compared gut microbiota from stool samples of gastric cancer patients in order to study changes in gut microbiota associated with these gastric cancer types/subtypes.

## Results

### Microbiota diversity

#### Alpha-diversity

**Fig. 1:**
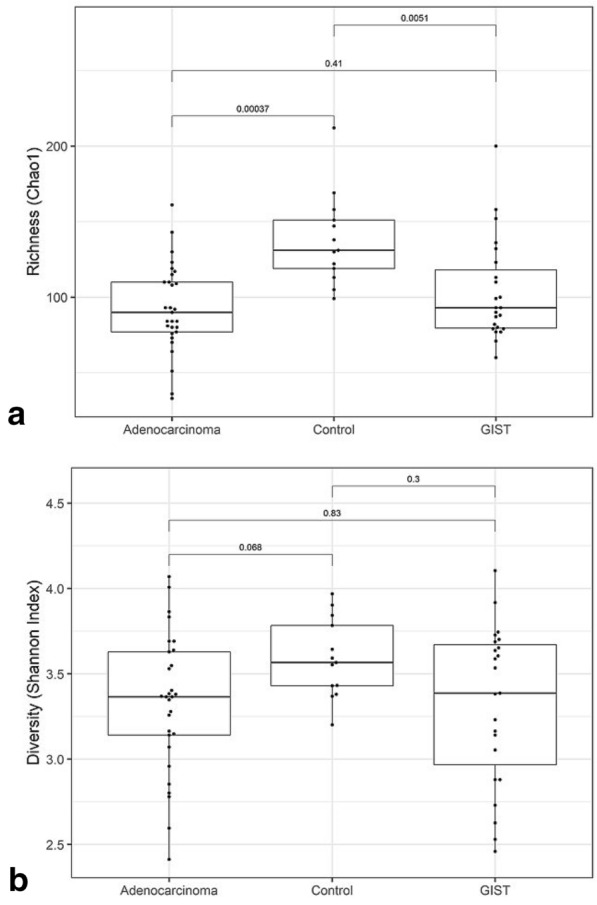
Gut microbiota richness and diversity in cancer patients and controls. Comparison of microbiota. **a** Richness (Chao1) and **b** diversity (Shannon index) in stool samples of patients with gastric adenocarcinoma, GIST and controls. The adjusted p-values are shown for each pairwise comparison

**Fig. 2:**
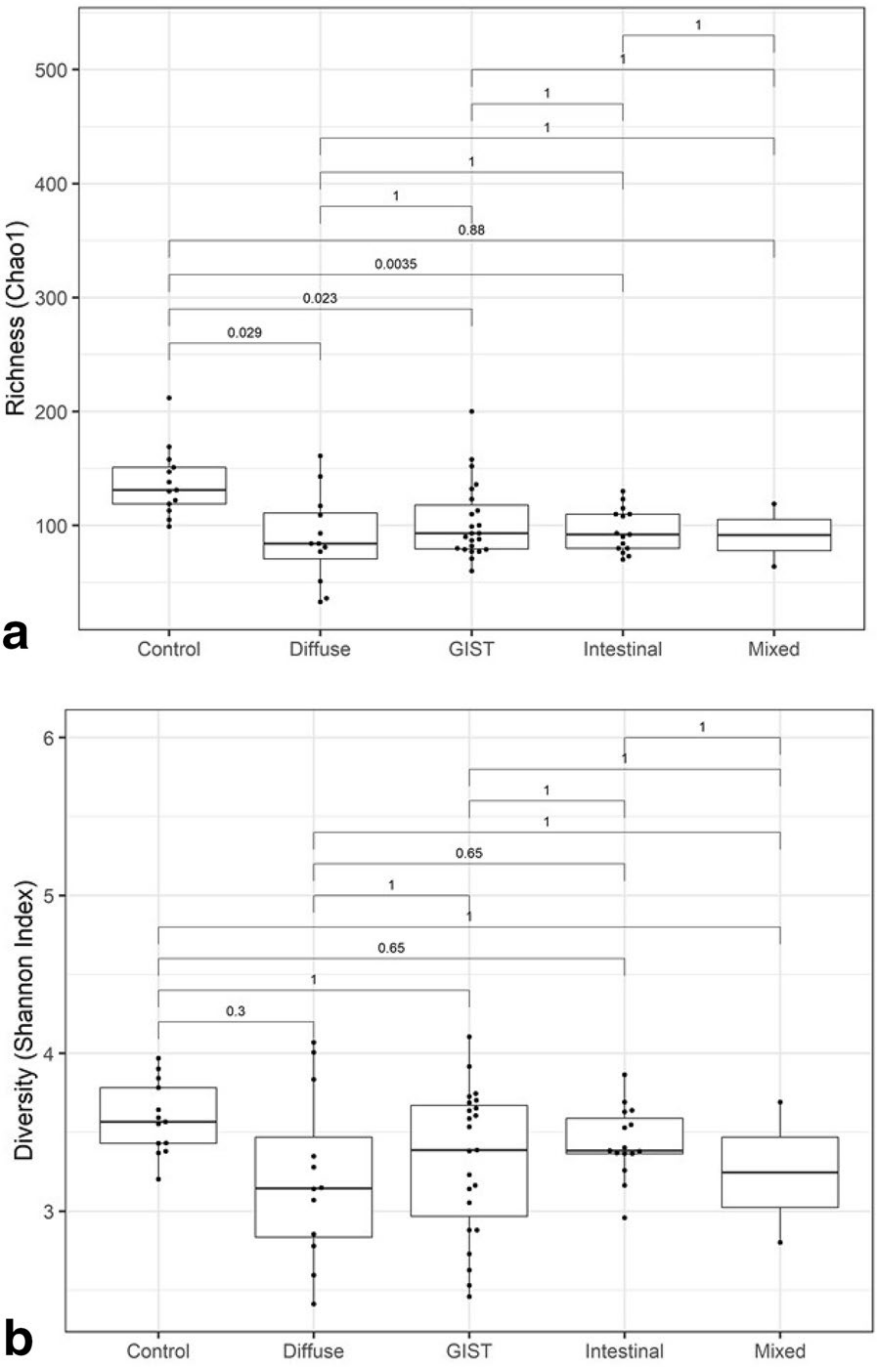
Gut microbiota richness and diversity in gastric cancer sub-groups. Comparison of microbiota. **a** Richness (Chao1) and **b** diversity (Shannon index) in stool samples of patients with diffuse gastric adenocarcinoma, intestinal gastric adenocarcinoma, mixed gastric adenocarcinoma, GIST and controls. The adjusted p-values are shown for each pairwise comparison

Gut microbiota richness and diversity were studied for different gastric cancer groups (adenocarcinoma and GIST) and the results (Fig. 1) showed lower microbiota richness and diversity in patients with gastric adenocarcinoma and gastric GIST patients compared to controls. We observed significant differences in microbiota richness between cancer patients and controls (Chao1; Fig. 1a). A similar trend was observed in microbiota diversity but this was not significant (Shannon index; Fig. 2b). No significant differences were observed between adenocarcinoma and GIST patients for richness (Fig. 1a) or diversity (Fig. 1b).

Patients with diffuse adenocarcinoma had the lowest microbiota diversity amongst the gastric cancer subgroups (diffuse, intestinal, mixed & GIST) (Fig. 2). Although all cancer subgroups had lower alpha diversity and a significantly lower microbiota richness (Chao1; Fig. 2a) compared to controls, the differences were not significant for diversity (Shannon index; Fig. 2b).

#### Beta-diversity

**Fig. 3:**
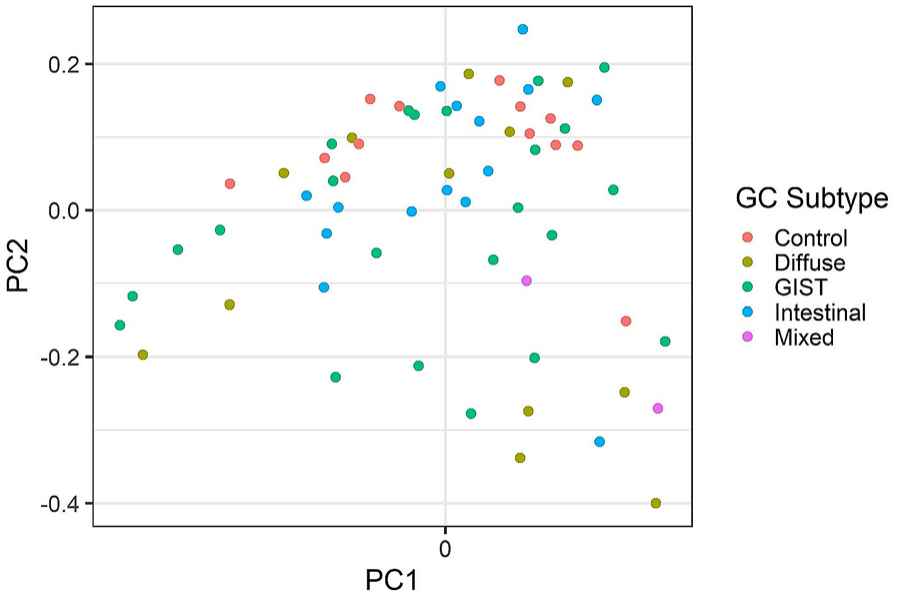
Microbiota community similarity between the samples. Principal coordinates analysis (PCoA) visualizes the variation in sample dissimilarity (Bray–Curtis beta-diversity based on genus-level bacterial profiles). The gastric cancer subtypes are indicated by color

Differences in microbiota composition were compared between controls and different GC groups and GC subgroups. The beta-diversity showed significant differences in microbiota composition between controls and adenocarcinoma (p = 0.03), and between controls and different adenocarcinoma subgroups (Table 1). However, no significant differences in microbiota composition were observed between the adenocarcinoma subgroups (Table 1, Fig. 3).

**Table 1:** Beta diversity analysis (PERMANOVA) for differences in gut microbiota between different gastric cancer sub-groups

Groups compared	R2	Adjusted p-value
Control *vs* Diffuse adenocarcinoma	0.08	0.05
Control *vs* GIST	0.05	0.05
Control vs Intestinal adenocarcinoma	0.07	0.05
Control *vs* Mixed adenocarcinoma	0.13	0.05
Diffuse adenocarcinoma *vs* GIST	0.02	0.89
Diffuse *vs* Intestinal adenocarcinoma	0.04	0.58
Diffuse adenocarcinoma *vs* Mixed	0.07	0.66
GIST *vs* Intestinal adenocarcinoma	0.03	0.32
GIST *vs* Mixed adenocarcinoma	0.04	0.54
Intestinal vs Mixed adenocarcinoma	0.08	0.20

### Significant taxa with differential relative abundance in adenocarcinoma, GIST and control

In order to identify taxa that had significant differences in relative abundance in pair-wise comparisons between the groups, we performed the ALDEx2 differential abundance analysis (Table 2).

**Table 2:** Taxonomic groups with significant differences (p ≤ 0.05) in pair-wise comparison of gastric cancer types

Taxa	Adjusted p-value	Effect^a^	Groups compared
Family			
*Lactobacillaceae*	< 0.01	0.64	Adenocarcinoma *vs* Control
*Enterobacteriaceae*	< 0.01	− 1.22	Adenocarcinoma *vs* Control
*Oscillospiraceae*	0.01	0.64	Adenocarcinoma *vs* Control
*Bifidobacteriaceae*	0.03	0.85	Adenocarcinoma *vs* Control
*Eubacteriaceae*	0.04	0.71	Adenocarcinoma *vs* Control
*Lactobacillaceae*	0.03	− 0.75	Control *vs* GIST
*Enterobacteriaceae*	0.03	1.01	Control *vs* GIST
Genus			
*Oscillibacter*	< 0.01	1.21	Adenocarcinoma vs Control
*Lachnoclostridium*	< 0.01	0.88	Adenocarcinoma vs Controls
*Bifidobacterium*	0.01	0.91	Adenocarcinoma vs Controls
*Parabacteroides*	0.03	0.60	Adenocarcinoma vs Controls
*Barnesiella*	0.05	0.62	Adenocarcinoma vs Controls
*Oscillibacter*	< 0.01	− 1.30	Control vs GIST

^a^ALDEx2 standardized effect size (see Methods)

**Fig. 4:**
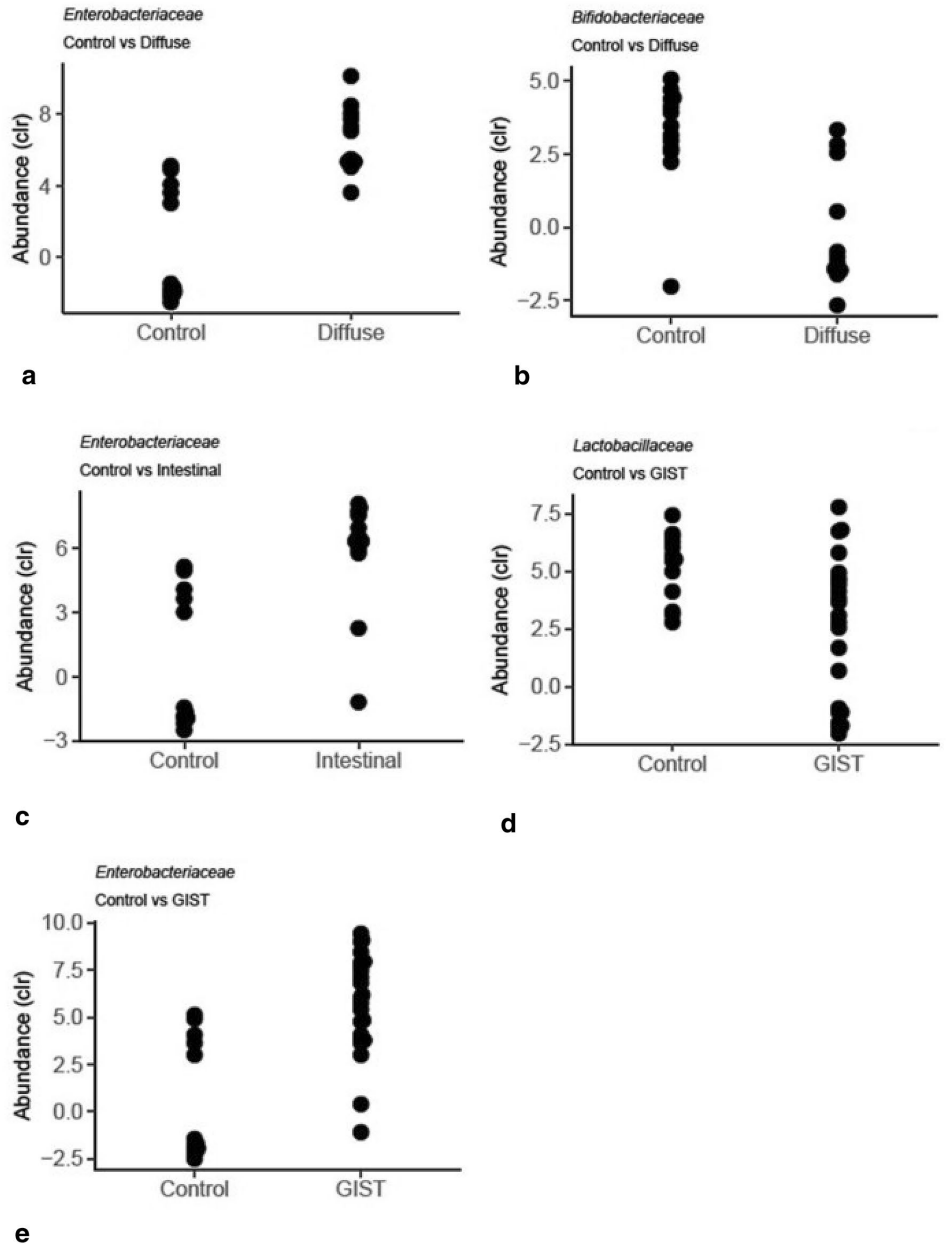
Significant taxa in paired group comparison. Taxa with significant differences (p ≤ 0.05; ALDEx2) in pair-wise comparison between gastric cancer subgroups and controls

Higher abundance of bacteria belonging to *Enterobacteriaceae* and lower abundance of those belonging to *Lactobacillaceae* was commonly observed in adenocarcinoma and GIST patients (Table 2, Fig. 4) compared to controls. Additionally, lower abundance of *Oscillospiraceae, Bifidobacteriaceae,* and *Eubacteriaceae* was seen in adenocarcinoma patients compared to controls.

At the genus level, significantly lower abundances of *Oscillibacter, Lachnoclostridium, Bifidobacterium, Parabacteroides* and *Barnesiella* were observed in adenocarcinoma patients compared to controls. In GIST patients, we observed a significantly lower abundance of *Oscillibacter* compared to controls.

### Significant taxa with differential relative abundance in diffuse, intestinal, mixed, GIST and controls

We also compared microbiota composition in patients grouped according to adenocarcinoma subgroups (diffuse, intestinal and mixed) and the taxa with significant differential abundance in various pair-wise group comparisons are shown in Table 3.

**Table 3:** Taxa with significant differences (p ≤ 0.05) in pair-wise comparison of gastric cancer subgroups

Taxa	Adjusted p-value	Effect^a^	Groups compared
Family			
*Enterobacteriaceae*	0.02	1.24	Control vs Diffuse adenocarcinoma
*Bifidobacteriaceae*	0.05	− 1.13	Control vs Diffuse adenocarcinoma
*Lactobacillaceae*	0.03	− 0.76	Control vs GIST
*Enterobacteriaceae*	0.03	1.02	Control vs GIST
*Enterobacteriaceae*	0.04	1.15	Control vs Intestinal adenocarcinoma
Genus			
*Oscillibacter*	< 0.01	− 1.40	Control vs GIST
*Oscillibacter*	0.01	− 1.26	Control vs Intestinal adenocarcinoma
*Holdemania*	0.02	1.75	Control vs Mixed adenocarcinoma
*Holdemania*	< 0.01	1.39	GIST vs Mixed adenocarcinoma
*Holdemania*	0.03	1.21	Intestinal vs Mixed adenocarcinoma

^a^ALDEx2 standardized effect size (see Methods)

Significantly higher abundance of *Enterobacteriaceae* was seen in both of the adenocarcinoma subgroups; diffuse (Fig. 4a) and intestinal (Fig. 4c), and in GIST (Fig. 4e) compared to the controls, while significantly lower abundance of *Bifidobacteriaceae* was seen only in the diffuse subgroup (Fig. 4b). The genera with significant differential abundance in cancer subgroups were: *Oscillibacter*, having lower abundance in intestinal subgroup and in GIST compared to control, and *Holdemania,* having higher abundance in mixed subgroup compared to controls and also compared to GIST and intestinal subgroup.

## Discussion

Adenocarcinoma of stomach is associated with gastric inflammation and *H. pylori* infection [12] that changes the stomach environment and can subsequently have an effect on the composition of the intestinal microbiota. On the other hand, intestinal microbiota plays an important role in the host immunity and inflammation. There are very few studies regarding changes in the intestinal microbiota in patients with gastric tumors, although changes in gastric microbiota have been reported previously [13]. Moreover, there are no gut microbiota studies, to the best of our knowledge, that have been carried out in different subtypes of gastric adenocarcinoma (diffuse and intestinal) and GISTs, which are quite different histologically and pathologically.

Our microbiota analysis from stool samples showed lowest alpha diversity and richness in patients with gastric adenocarcinoma, followed by patients with gastric GIST, and highest in controls. The alpha diversity (Shannon index) in adenocarcinoma and gastric GIST patients was however not significantly lower compared to controls (Fig. 1b) although the differences were significant for richness (Chao1, Fig. 1a). When analyzed separately, diffuse subtype had the lowest microbiota diversity, followed by intestinal subtype of adenocarcinoma and then gastric GIST (Fig. 2), although the diversity (Shannon index) was not significantly lower than controls. On the other hand, beta-diversity analysis showed that all cancer subgroups had significant differences in microbiota composition compared to controls (Table 1). A progressive decrease in gastric microbiota richness has been reported in premalignant GC stages with significant differences seen in intraepithelial neoplasia and GC compared to control [13, 14], while no significant changes in stool microbiota diversity is reported in patients with gastritis or metaplasia and controls [15].

In our study, the healthy controls were of younger age compared to the patients which could have confounding effect on the microbiota diversity results. However, among the cancer subgroups, patients with diffuse adenocarcinoma had lower microbiota diversity compared to intestinal adenocarcinoma patients, although the average age of patients with diffuse types was lower (69 years) than those with intestinal type (75 years). The lower microbiota diversity in diffuse adenocarcinoma thus seems to be more related to its undifferentiated cell morphology and aggressive tumor characteristics.

Gut bacteria are reported to affect immune response in tumor environment. A reduced gut microbiota diversity in breast cancer patients is linked to low immune infiltrate in breast tumors [16]. The diffuse subtype of gastric adenocarcinoma is characterized by undifferentiated cell phenotype, is highly invasive, and has poor immune cells infiltration, while both GIST [17] and intestinal adenocarcinoma have higher immune cells infiltration compared to diffuse type [18]. Poor tumor immune infiltrate, common in diffuse type, might be linked to the lower gut microbiota diversity seen in these patients in our study. The other reason could be that 58% of patients with diffuse, compared to 36% with intestinal type in our study had metastatic disease and the lower diversity might as well be related to advanced stage of cancer in diffuse type. Moreover, only diffuse subtype had significantly low abundances of bacteria belonging to *Bifidobacteriaceae* family*. Bifidobacterium,* an important member of this family and widely used as probiotic, is reported to suppress metastasis in mouse model via its effect on IL-11 expression and subsequent effect on circRNA/microRNA/sox9 axis and epithelial-mesenchymal transition genes [19]. Abundance of gut *Bifidobacterium* is also associated with the accumulation of activated antigen-specific T cells in the tumor microenvironment and its low abundance is related to more aggressive tumors in mice [20]. This is similar to our observation of lower abundances of *Bifidobacteriaceae* in diffuse adenocarcinoma, which have low immune cell infiltrate and are also more aggressive. Moreover, oral administration of *Bifidobacterium* is reported to control tumor growth in mice by increasing T cell accumulation in tumor microenvironment and increasing the efficacy of programmed cell death protein 1 (PD-L1) specific antibody therapy [20]. The abundance of gut *Bifidobacterium* thus seems to have an effect on tumor growth via its influence on host immunity and administration of *Bifidobacterium* supplements could have beneficial effects in cancer patients.

Significantly higher abundances of *Enterobacteriaceae* compared to controls were common in all cancer subgroups i.e. diffuse and intestinal adenocarcinoma and GIST. Increased abundance of gut *Enterobacteriaceae* is strongly related to gut inflammation, which reprograms their metabolism to confers them growth advantage over other bacteria to survive in inflammatory environment [21]. Recently, association between increased *Enterobacteriaceae* abundance and long-term mortality risk is reported in a large Finnish population cohort [22]. Abundance of gut *Enterobacteriaceae* is also reported to increase with the severity of premalignant gastric cancer stages, with significantly increased abundances at metaplasia stage [15]. Similar to our results, higher abundance of gut *Enterobacteriaceae* is reported in preoperative Chinese gastric cancer patients compared to healthy controls [23]. However, low abundance of *Bacteroidaceae* in gastric cancer patients reported in this study [23] was not observed in our samples, while adenocarcinoma and GIST patients in our study had significantly low abundance of *Lactobacillaceae*. *Lactobacillus* a member of this family and a probiotic is one of the dominant bacteria in fermented dairy products especially yogurts, the intake of which is associated with a reduced risk of many types of cancer [24].

At the genus level, low abundances of *Oscillibactor, Lachnoclostridium, Bifidobacterium, Parabacteroides* and *Barnesiella* were seen in overall adenocarcinoma patients compared to controls. Similar to our findings, low abundances of gut *Parabacteroides* and *Barnesiella* is reported to be associated with the increasing severity of gastric lesions [15], while low abundances of Bifidobacteria*,* Lactobacilli, and higher abundances of *Escherichia coli,* Staphylococci, Enterococci and Peptostreptococci are reported in gastric cancer patients [25].

To the best of knowledge, our study is the first to analyze gut microbiota in GIST patients. GISTs are thought to arise from interstitial cells of Cajal that control the muscular movement of the gastrointestinal tract. Other than the abdominal pain and non-specific symptoms, bleeding and obstruction are among the commonly reported symptoms in GIST patients [26]. However, the role of microbiota is poorly documented. Probiotic treatment is reported to increase the number of Interstitial cells of Cajal in cats with chronic constipation [27]. Similarly, *Clostridium butyricum* has been found to regulate TLR2 expression in Interstitial cells of Cajal in ulcerative colitis [28]. We found that, alpha diversity in stool samples of GIST patients was not significantly different from that of controls. However, GIST patients had increased abundance of gut *Enterobacteriaceae* and reduced abundance of *Lactobacillaceae* and *Oscillibacter* compared to the controls, similar to that seen in gastric adenocarcinoma patients. *Oscillibacter* that showed lower abundance in GIST and intestinal adenocarcinoma in our study, is related to metabolic and inflammatory diseases, with lower levels seen in obese individuals [29] and high abundances in ulcerative colitis mice that correlate with serum interleukin IL6 and IL- levels [30]. Probiotics intake is reported to increase *Oscillibacter* abundance and changes gut microbiota composition that suppresses growth of hepatocellular carcinoma in mice model [31].

Higher abundance of *Holdemania* was seen in patients with mixed adenocarcinoma compared to the controls and also compared to the other cancer groups. Abundance of *Holdemania* is associated with heavy alcohol drinking [32], and with anxiety and stress [33], while a significant increased risk of gastric cancer with heavy drinking is reported [34]. As there were only two patients with mixed adenocarcinoma, it is difficult to draw any conclusions for this subgroup.

## Conclusion

Our study shows that higher abundance of *Enterobacteriaceae* is a common feature seen in all subtypes of cancer of the stomach; diffuse, intestinal and gastric GIST. Patients with diffuse adenocarcinoma have lower gut microbiota diversity which could be related to their more aggressive tumor type or advanced stage of tumor. Further studies would be helpful in evaluating their role as a marker of tumor progression.

## Methods

### Patients and sample collection

Stool samples were collected by clinicians (AK and PP) from Finnish patients with gastric cancer or GIST, at Surgical and Meilahti Hospitals in Helsinki Uusimaa Hospital District, Finland. Only those patients who had no antimicrobial medication during the last 6 months prior to sample collection and had not started any cancer treatment were included in the study. The patients included were: GIST (N = 23) and gastric adenocarcinoma (N = 29). Of these, sequencing data of 6 GIST and 25 adenocarcinoma patients from our previous study [35], that compared microbiota in patients based on location of tumor in gastrointestinal tract (stomach, colon and rectum), were included in the present study. For controls, stool samples of 13 healthy Finnish individuals were collected and their sequencing data used in previous studies [35, 36] were included in the present study. All samples were collected and processed for DNA isolation and next generation sequencing (NGS) following same protocol and in the same laboratory. The details of the patient groups and control included in the analysis is described in Table 4.

**Table 4:** Characteristics of patients and controls included in the gut microbiota analysis

Group	No of cases^a^	Average age (years)	Sex (M/F)
Gastric adenocarcinoma	29	72 ± 11	14/15
Diffuse adenocarcinoma	12	69 ± 11	5/7
Intestinal adenocarcinoma	15	75 ± 10	8/7
Mixed adenocarcinoma	2	69 ± 11	1/1
GIST	23	67 ± 14	11/12
Controls	13	44 ± 14	3/10

^a^Samples from previous study: Diffuse 11, Intestinal 13, Mixed 1, GIST 6, Controls 13

### 16S rRNA gene sequencing

DNA was extracted from stool samples using PSP Spin Stool DNA Plus Kit (Stratec Molecular, Berlin, Germany) and quality and quantity of DNA was checked by Qubit 3.0 Fluorimeter (Thermo Fisher Scientific, Waltham, MA, USA). Sequencing libraries were prepared from 3 ng of DNA, using Ion 16S Metagenomics kit (Thermo Fisher Scientific, Waltham, MA, USA) according to the vendor’s instructions. Six hypervariable regions (Primer set V2, V4, V8 and Primer set V3, V6-7, V9) of 16S rRNA gene were amplified in two reactions/sample. After PCR, the samples were end-repaired, purified with Agencourt® AMPure® XP beads (Beckman Coulter, Brea, California, USA) and ligated to barcoded sequencing adapters according to the kit protocol. The libraries were quantified by the TapeStation (Agilent Technologies, Santa Clara, CA, USA) and samples were diluted to a 10 pM concentration. The libraries were pooled and the template preparation was performed with either Ion OneTouch 2 system/ or Ion Chef system using the Ion PGM™ Hi-Q™ OT2 Kit/ Ion PGM™ Hi-Q™ Chef Kit (Thermo Fisher Scientific, Waltham, MA, USA) following the kit protocols. The OneTouch 2/ Ion Chef system was used for emulsion PCR and the quality of resulting Ion Spheres were checked with Qubit 3.0 fluorometer (Thermo Fisher Scientific, Waltham, MA, USA). Sequencing was performed on the Ion PGM system using the Ion 318™ Chip (Thermo Fisher Scientific, Waltham, MA, USA) and Ion PGM Hi-Q Sequencing kit (Thermo Fisher Scientific, Waltham, MA, USA).

### Data analysis

Raw data from 16S rRNA gene sequencing was processed for quality check and filtering using Ion Torrent Suite software (Thermo Fisher Scientific, Waltham, MA, USA). Data was further analyzed for operational taxonomic unit (OTU) clustering, taxonomic classification using IonReporter v.5.10 (Thermo Fisher Scientific, Waltham, MA, USA) with Metagenomics 16S pipeline w1.1, applying default settings, and using Curated MicroSEQ(R) 16S Reference Library v2013.1 and Curated Greengenes v13.5 databases. Consensus tables were created by summing up all read counts from different V regions with identical taxonomic rank detection. A summary statistics of sample data is provided in Additional file 1: Table S1.

Data of 65 stool samples (including 44 published earlier) were grouped as Adenocarcinoma, GIST and Controls for cancer group comparison and as Intestinal, Diffuse, Mixed, GIST and Controls for cancer subgroup comparison. Alpha diversity (Shannon index) and richness (Chao1) of the gut microbiota among different groups was studied at the genus level using the *microbiome* [37] and *vegan* R packages [38]. The differences in the group comparison were tested for significance by the Wilcoxon-Mann–Whitney testand corrected for multiple testing with the Benjamini–Hochberg FDR method [39].

Unsupervised principal coordinates analysis (PCoA) was conducted with the *phyloseq* R package [40] and based on Bray–Curtis dissimilarity index [41]. Only the genera that were detected in at least 20% of all samples were included in the analysis. We used ALDEx2 [42] to identify taxonomic groups that showed significant (p <  = 0.05) differences in paired group comparisons. The ALDEx2 R implementation takes advantage of the clr transformation for relative abundances to remove compositionality bias, and provides empirical p-values with Benjamini–Hochberg FDR correction. The standardized effect size and p-value estimation is based on a probabilistic sampling procedure described in [42]. In summary, the standardized effect size refers to the between group difference, scaled by the maximum within group difference.

## Supplementary Information

**Additional file 1: Table S1.** Summary statistics of the sample data.

## Data Availability

The datasets used and/or analysed during the current study are available from the corresponding author on reasonable request.
